# KAP related to first aid among first-year ethnic minority students: a cross-sectional study using latent profile analysis

**DOI:** 10.3389/fpubh.2026.1873238

**Published:** 2026-07-23

**Authors:** Xiulan Yang, Jie Yang, Weiyan Tian, Youyong Peng, Liu Yang, Xu Ren, Mingtao Quan, Jing He, Mei Chen

**Affiliations:** 1 Department of Emergency Medicine, Affiliated Hospital of Zunyi Medical University, Zunyi, Guizhou, China; 2 School of Nursing, Zunyi Medical University, Zunyi, Guizhou, China; 3 Department of Emergency Medicine, Third Affiliated Hospital of Zunyi Medical University (The First People’s Hospital of Zunyi), Zunyi, Guizhou, China; 4 Department of Nursing, Affiliated Hospital of Zunyi Medical University, Zunyi, Guizhou, China

**Keywords:** China, ethnic and racial minorities, first aid, health education, latent class analysis, student health services

## Abstract

**Objective:**

First aid-related knowledge, attitudes, and self-reported practices are important components of emergency preparedness among university students. However, evidence on first aid-related KAP patterns among ethnic minority freshmen in China remains limited. This study aimed to assess first aid-related knowledge, attitudes, and self-reported practices, identify latent profiles, and examine factors associated with profile membership among ethnic minority freshmen at a medical university in Guizhou Province, China.

**Methods:**

A university-based cross-sectional study was conducted in September 2025 among first-year students at a medical university in Guizhou Province, China, prior to the implementation of a first-aid education program. During compulsory military training, questionnaires were administered to all first-year students through administrative training units. Ethnic minority participants were identified *post hoc* based on self-reported demographic information. After exclusion of invalid responses according to predefined quality criteria, 805 valid questionnaires from ethnic minority freshmen were included in the final analysis. A first aid-related knowledge, attitude, and practice questionnaire was used. Knowledge scores were rescaled to a 1–5 metric, while attitude and practice scores were calculated as mean item scores. Latent profile analysis was applied to identify unobserved subgroups with distinct KAP patterns. Associations between profile membership and potential predictors were examined using univariable and multivariable binary logistic regression models.

**Results:**

Two latent profiles were identified: a high-attitude/high-practice profile (18.6%) and a low-attitude/low-practice profile (81.4%). Transformed knowledge scores differed only slightly between profiles, whereas attitudes and self-reported practices showed clearer differences. In the multivariable model, previous non-avoidance of providing first aid, attending first-aid training three or more times, and certificate- or credit-related training willingness were associated with membership in the high-attitude/high-practice profile.

**Conclusion:**

Substantial heterogeneity was observed in first aid-related attitudes and self-reported practices among ethnic minority freshmen, with most students classified into the low-attitude/low-practice profile. Previous non-avoidance of providing first aid, attending first-aid training three or more times, and certificate- or credit-related training willingness were associated with membership in the high-attitude/high-practice profile. The findings suggest that university first-aid education should move beyond knowledge transmission and place greater emphasis on repeated, scenario-based, and practice-oriented training. Because actual first-aid competence was not objectively assessed, these findings should be interpreted as reflecting differences in first aid-related attitudes and self-reported practices rather than actual emergency response capability.

## 1 Introduction

First aid is an essential component of public emergency response and can reduce preventable harm before professional medical care becomes available (1). Many high-income countries place a strong emphasis on public first-aid training and have incorporated it into national education systems. For example, approximately 80% of the population in Germany has received formal first-aid training (2), and nationwide surveys in Norway have likewise reported broad first-aid training coverage (3). In contrast, the population coverage of emergency first-aid knowledge and skills in China is estimated to be only about 1%, far below that in high-income settings, which may undermine the effectiveness of prehospital emergency care (4). The Healthy China Action (2019–2030) explicitly set a target of increasing the proportion of individuals holding first-aid training certificates to at least 3% by 2030 (5).

University students represent an important population for first-aid education because they are often exposed to campus, community, and public-space emergencies, and may become immediate bystanders in time-sensitive situations (6). However, students’ first aid-related preparedness remains uneven, and knowledge acquisition alone may not be sufficient to ensure willingness or readiness to act in emergencies (7). In many institutions, first-aid education remains largely theory-based; although students may acquire some conceptual knowledge, they often receive insufficient hands-on training and may therefore be reluctant to intervene in real-life emergencies (8). In addition, first-aid education varies across regions and student backgrounds. Students from rural areas or ethnic minority groups may have fewer opportunities to receive first-aid training and consequently tend to report lower levels of first-aid knowledge and skills (9, 10). Guizhou Province is home to a large ethnic minority population, and many ethnic minority freshmen enrolled in local universities come from underdeveloped rural or mountainous areas where access to systematic first-aid training before university entry may be limited (11).

The knowledge–attitude–practice (KAP) framework has been widely used in health-related behavior research (12); however, it should not be interpreted as a simple linear process in which knowledge directly leads to behavioral change. In first-aid contexts, individuals’ willingness to provide assistance and actual helping behavior are influenced by multiple psychological and contextual factors, including self-efficacy, perceived behavioral control, subjective norms, emotional responses, fear of causing harm, and environmental constraints, which are consistent with the Theory of Planned Behavior, Social Cognitive Theory, and self-efficacy theory. Despite its widespread application, traditional variable-centered analyses based on the KAP framework typically focus on average associations and may overlook substantial heterogeneity within populations. Latent profile analysis (LPA), a person-centered approach, enables the identification of unobserved subgroups by classifying individuals according to similar response patterns across multiple observed indicators, thereby providing a more nuanced understanding of behavioral heterogeneity (13). Against this background, the present study applied a KAP-based questionnaire to assess first aid-related knowledge, attitudes, and self-reported practices among ethnic minority freshmen at a medical university in Guizhou, China, and subsequently used LPA to explore whether distinct latent subgroups existed with different KAP patterns, such as relatively favorable versus less favorable profiles. This approach aimed to better capture within-group heterogeneity and provide evidence for more targeted and tailored first-aid education strategies in university settings.

## 2 Methods

### 2.1 Study design and participants

A university-based cross-sectional survey was conducted in September 2025 among first-year students at a medical university in Guizhou Province, China, before the implementation of a first-aid education program. In China, compulsory military training is a standardized component of undergraduate education and provides a structured setting for accessing the entire first-year student cohort. During this period, questionnaires were administered to first-year students through administrative units responsible for organizing military training activities.

Ethnic minority participants were identified *post hoc* based on self-reported demographic information. After exclusion of responses that did not meet predefined data quality criteria, 805 valid questionnaires from ethnic minority freshmen were included in the final analysis. Eligible participants were first-year students enrolled in 2025 who provided electronic informed consent and completed the questionnaire. Exclusion criteria included missing key variables, logical inconsistencies, duplicate submissions, completion times under 5 min, or highly patterned responses.

The questionnaire consisted of 62 items, including 40 core first aid-related knowledge, attitude, and practice (KAP) items used for the main analyses. Sample size adequacy was evaluated using the commonly used subject-to-item ratio approach for questionnaire-based studies involving psychometric assessment (14, 15). Based on a ratio of 5–10 participants per item, 200–400 participants were required for the 40 core first aid-related KAP items, increasing to 250–500 after allowing for an estimated 20% invalid response rate. The final analytic sample of 805 participants exceeded these recommended ranges, indicating sufficient sample size for the planned analyses. No *a priori* power calculation was performed, and this assessment reflects sample adequacy rather than formal statistical power estimation.

### 2.2 Survey instrument

The initial questionnaire was developed on the basis of a literature review, first-aid education content, and expert consultation. Content validity and internal consistency were assessed before the main analysis.

The questionnaire was developed through a systematic process: ① The research team conducted a systematic review of the literature and relevant first-aid guidelines to establish the core dimensions of the questionnaire and generate an initial pool of items. The attitude domain focused on psychological constructs, including self-efficacy, a sense of social responsibility, and recognition of the first-aid training system. The practice domain distinguished between the willingness to provide direct first aid and indirect behavioral intentions, such as participating in training or public education activities. ② An expert panel review was conducted to assess and refine the content validity of the items. Fifteen experts in emergency medicine and emergency nursing evaluated the relevance, clarity, and comprehensibility of each item. Based on their feedback, wording and structure were optimized, resulting in a content validity index of 0.821. ③ A pilot survey (*n* = 81) was conducted before the formal investigation to preliminarily assess reliability and structural validity. The overall Cronbach’s *α* coefficient of the questionnaire was 0.875, indicating good internal consistency. A revised version of the questionnaire was subsequently developed based on the previous version. In the present study sample, further testing demonstrated good internal consistency for both the attitude and practice domains (Cronbach’s *α* > 0.85). Supplementary exploratory and confirmatory factor analyses were also conducted to provide preliminary evidence of structural validity, and the detailed results are reported in Supplementary Table S1.

The questionnaire included demographic characteristics and three first aid-related KAP domains: knowledge, attitude, and practice. The knowledge domain was measured using dichotomous items, whereas the attitude and practice domains were measured using Likert-type items. For latent profile analysis, knowledge scores were transformed to a 1–5 scale using the formula 1 + 4 × proportion correct. Mean item scores were calculated for the attitude and practice domains. This transformation was used only to place the three domain-level indicators on a common scale for latent profile modeling and did not imply that knowledge, attitude, and practice were psychometrically equivalent constructs. In this study, practice refers to self-reported first-aid-related behavioral intentions rather than objectively assessed first-aid performance.

### 2.3 Data collection

Data were collected using the Wenjuanxing online survey platform. Participants accessed the questionnaire by scanning a QR code. An electronic informed consent or assent statement was presented on the first page of the questionnaire, and only students who agreed to participate were allowed to proceed. The survey was anonymous, and all data were collected solely for research purposes.

### 2.4 Quality control

Several measures were taken to ensure data quality. First, all questionnaire items were set as mandatory, and skip logic was applied where appropriate to minimize missing data and improve response consistency; consequently, no item-level missing data were observed in the final dataset. Second, before questionnaire completion, researchers provided standardized instructions on the study purpose, response procedures, and survey requirements, without influencing participants’ answers. Third, after data collection, all questionnaires were screened according to predefined quality control criteria, and responses not meeting these criteria were excluded from the final dataset. This study is reported in accordance with the STROBE statement for cross-sectional studies.

### 2.5 Ethical considerations

This study was approved by the Ethics Committee of the Affiliated Hospital of Zunyi Medical University, and the consent procedures were reviewed and approved by the ethics committee. All participants provided electronic informed consent or assent before completing the anonymous questionnaire. The survey was anonymous, and no personally identifiable information was collected. Participation was voluntary, and participants could refuse to participate or withdraw from the survey at any time without providing a reason. Because the questionnaires were distributed during the military-training period, we specifically clarified that refusal to participate or withdrawal from the survey would not affect students’ military training, academic evaluation, access to university activities, or relationships with teachers, trainers, or administrators. The data were used only for research purposes and were analyzed in aggregate form.

### 2.6 Statistical analysis

Descriptive statistics and regression analyses were performed using SPSS version 29.0, and latent profile analysis was conducted using Mplus version 8.3. Categorical variables were presented as frequencies and percentages. Group comparisons were performed using Pearson’s chi-square test, and Fisher’s exact test was used when expected cell counts were small. Cramer’s V was calculated as an effect-size measure for categorical comparisons. Additional structural validity analyses were conducted as supplementary analyses. The full sample was randomly divided into two subsets: subset A for exploratory factor analysis and subset B for confirmatory factor analysis. The Kaiser–Meyer–Olkin value and Bartlett’s test of sphericity were used to assess the suitability of the data for factor analysis. In the exploratory factor analysis, factor extraction was guided by eigenvalues greater than 1 and the scree plot. In the confirmatory factor analysis, Likert-type items were treated as ordinal indicators, and the diagonally weighted least squares estimator was used. Detailed results are presented in Supplementary Table S1. Models with one to five latent profiles were fitted sequentially using the three domain-level indicators of knowledge, attitude, and practice. The optimal number of profiles was selected based on a combination of statistical fit indices, entropy, classification quality, class size, parsimony, model stability, and substantive interpretability, rather than on a single statistical test. The fit indices included the Akaike information criterion, Bayesian information criterion, sample-size-adjusted Bayesian information criterion, entropy, Lo–Mendell–Rubin likelihood ratio test, and bootstrap likelihood ratio test. Lower AIC, BIC, and aBIC values indicated better relative model fit, whereas entropy values closer to 1 indicated better classification accuracy. In addition to statistical fit indices, candidate profile solutions were evaluated according to class size, model stability, and substantive interpretability. Profiles were interpreted based on the combined patterns of knowledge, attitude, and practice scores, such as relatively favorable or less favorable attitude and self-reported practice patterns. Solutions with very small classes or unclear conceptual meaning were considered less suitable for explaining KAP heterogeneity.

Univariable binary logistic regression was used to estimate crude odds ratios, and multivariable binary logistic regression was used to estimate adjusted odds ratios. Latent profile membership was used as the dependent variable, with the low-attitude/low-practice profile coded as 0 and the high-attitude/high-practice profile coded as 1. The coding of independent variables used in the regression analyses is presented in Table 1. Odds ratios greater than 1 indicated higher odds of belonging to the high-attitude/high-practice profile. A two-sided *p* value < 0.05 was considered statistically significant.

**Table 1:** Coding of independent variables used in the binary logistic regression analyses.

Independent variable	Coding
Sex	0 = male, 1 = female
Age	0 = <18 years, 1 = 18–24 years
Medical-related educational background	0 = yes, 1 = no
Household registration	0 = urban, 1 = rural
Parents engaged in healthcare-related occupations	0 = yes, 1 = no
Previous avoidance of providing first aid	0 = yes, 1 = no
Whether willingness to participate in training would increase if certificates/academic credits were offered	0 = significantly increase, 1 = slightly increase, 2 = no change
First-aid training experience (four categories)	0 = no training, 1 = once, 2 = twice, 3 = ≥3 times

First-aid training experience was coded by combining previous first-aid training status and the number of training sessions attended.

## 3 Results

### 3.1 Descriptive results of knowledge, attitudes, and practices

**Figure 1:**
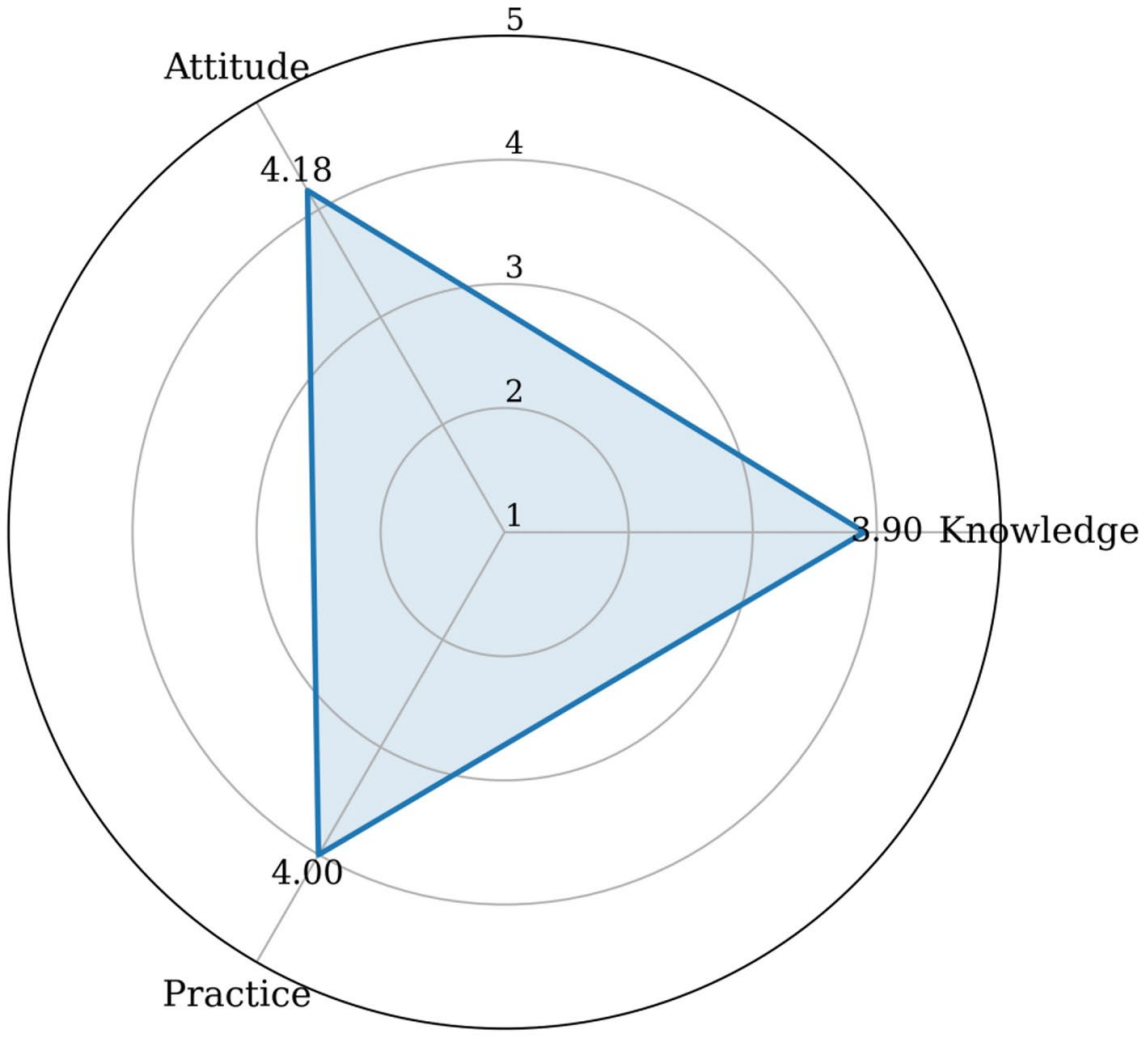
Overall knowledge, attitude, and practice scores among ethnic minority freshmen.

Before latent profile analysis, the overall KAP scores were examined. The mean transformed knowledge, attitude, and practice scores were 3.90 ± 0.36, 4.18 ± 0.52, and 4.00 ± 0.50, respectively. As shown in Figure 1, attitude had the highest score, whereas knowledge had the lowest. These descriptive findings were further examined using latent profile analysis to identify subgroups with distinct KAP patterns.

### 3.2 Model fit results of the latent profile analysis among ethnic minority freshman students

Models with one to five latent profiles were fitted sequentially. The selection of the optimal model was based on statistical fit indices, classification quality, class size, parsimony, model stability, and substantive interpretability, rather than on a single statistical test. Although AIC, BIC, and aBIC decreased as the number of profiles increased, this pattern alone was not considered sufficient to justify retaining more complex models.

The two-profile model showed acceptable classification quality, with an entropy value of 0.858, and produced two substantively meaningful groups with reasonable class proportions (18.63 and 81.37%). The three-profile solution did not provide a clear improvement over the two-profile model, as both the LMR test and the BLRT were not statistically significant. In addition, the three-profile solution did not yield a clearer or more theoretically meaningful interpretation of the KAP patterns. Although the four- and five-profile solutions showed lower information criteria, they generated very small classes, including classes accounting for only 0.50% of the sample, suggesting possible over-extraction and limited model stability.

**Figure 2:**
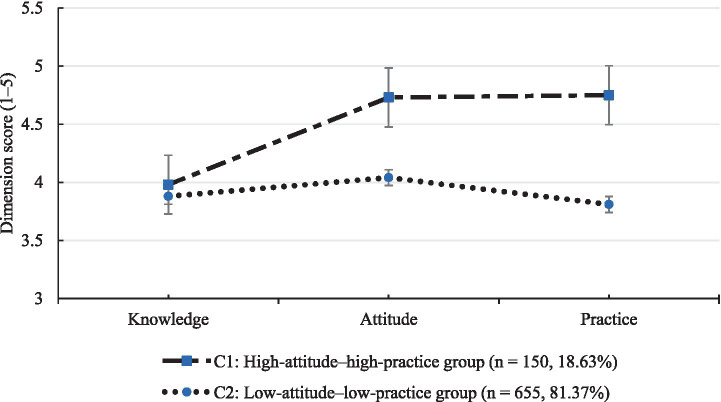
Comparison of scores on the knowledge, attitude, and practice dimensions between the two latent profiles.

Therefore, the two-profile solution was retained as the most parsimonious, stable, and interpretable model. The two profiles were labeled according to their score patterns. C1 was labeled the high-attitude/high-practice profile and included 150 students (18.6%), whereas C2 was labeled the low-attitude/low-practice profile and included 655 students (81.4%). The profiles differed mainly in attitude and self-reported practice scores, while the difference in transformed knowledge scores was relatively small. Thus, the two-profile solution should be interpreted primarily as reflecting differences in attitudes and self-reported practices rather than marked differences in knowledge (Figure 2 and Table 2).

**Table 2:** Potential profile model fitting indices.

Category	AIC	BIC	aBIC	Entropy	LMR (*P*-value)	BLRT (*P*-value)	Class probability (%)
1	3036.93	3065.07	3046.02	–	–	–	–
2*	2734.73	2781.64	2749.88	0.858	<0.05	<0.05	18.63/81.37
3	2589.40	2655.07	2610.61	0.874	0.39	0.40	8.32/72.92/18.76
4	2443.24	2527.68	2470.52	0.899	<0.05	<0.05	10.56/18.76/0.50/70.19
5	2327.69	2430.89	2361.02	0.889	<0.05	<0.05	0.50/3.11/25.60/16.52/54.29

AIC = Akaike information criterion; BIC = Bayesian information criterion; aBIC = sample-size-adjusted Bayesian information criterion; LMR = Lo–Mendell–Rubin likelihood ratio test; BLRT = bootstrap likelihood ratio test; *indicates the optimal profile solution.

### 3.3 Comparison of general characteristics and first-aid training-related variables between the two latent profiles among ethnic minority freshman students

A total of 822 questionnaires were returned, of which 17 were excluded as invalid, leaving 805 ethnic minority freshman students for the final analysis. In the overall sample, 234 participants (29.1%) were male and 571 (70.9%) were female. Most participants were aged 18–24 years (764, 94.9%). In total, 121 participants (15.0%) were enrolled in medical-related majors, and 656 (81.5%) had rural household registration. Overall, 378 participants (47.0%) had previously received first-aid training, 32 (4.0%) had parents working in healthcare-related occupations, 48 (6.0%) had previously avoided providing first aid, and 788 (97.9%) expressed a willingness to participate in first-aid training. The two profiles differed significantly in previous first-aid training, the number of training sessions attended, previous avoidance of providing first aid, and certificate- or credit-related training willingness. The corresponding Cramer’s *V* values indicated small effect sizes. No significant differences were observed in sex, age, medical-related educational background, household registration, parental healthcare-related occupation, or willingness to participate in first-aid training (Table 3).

**Table 3:** Comparison of general characteristics and first-aid training-related variables between the two latent profiles among ethnic minority freshmen.

Variable	Category	Total	C1High-attitude–high-practice group (*n* = 150)	C2Low-attitude–low-practice group (*n* = 655)	Test statistic	*p*-value	Cramer’s *V*
Sex	Male	234	38(25.3)	196(29.9)	1.247	0.264	0.039
	female	571	112(74.7)	459(70.1)			
Age	<18 years	41	9(6.0)	32(4.9)	0.314	0.575	0.020
	18–24 years	764	141(94.0)	623(95.1)			
Medical-related major	Yes	121	26(17.3)	95(14.5)	0.765	0.382	0.031
	No	684	124(82.7)	560(85.5)			
Household registration	Urban	149	35(23.3)	114(17.4)	2.844	0.092	0.059
	Rural	656	115(76.7)	541(82.6)			
Previous first-aid training	Yes	378	88(58.7)	290(44.3)	10.149	0.001	0.112
	No	427	62(41.3)	365(55.7)			
Number of training sessions attended	Once	106	17(19.3)	89(30.7)	8.475	0.014	0.150
	Twice	103	20(22.7)	83(28.6)			
	Three or more times	169	51(58.0)	118(40.7)			
Parents engaged in healthcare-related occupations	Yes	32	6(4.0)	26(4.0)	0.00	0.986	0.001
	No	773	144(96.0)	629(96.0)			
Previous avoidance of providing first aid	Yes	48	2(1.3)	46(7.0)	7.046	0.008	0.094
	No	757	148(98.7)	609(93.0)			
Willingness to participate in first-aid training	Yes	788	149(99.3)	639(97.6)	Fisher	0.22	0.048
	No	17	1(0.7)	16(2.4)			
Whether willingness would increase if certificates or academic credits were offered	Significantly increase	611	119(79.3)	492(75.1)	17.95	<0.001	0.149
	Slightly increase	167	19(12.7)	148(22.6)			
	No increase	27	12(8)	15(2.3)			

Data are presented as *n* (%). C1 represents the high-attitude/high-practice profile, and C2 represents the low-attitude/low-practice profile. The variable “number of training sessions attended” was calculated only among participants who had previously received first-aid training.

### 3.4 Univariable and multivariable binary logistic regression analyses of latent profile membership among ethnic minority freshmen

Latent profile membership was used as the dependent variable. Sex, age, medical-related educational background, household registration, whether the parents were engaged in healthcare-related occupations, previous avoidance of providing first aid, first-aid training experience (four categories), and whether willingness to participate in training would increase if certificates or academic credits were offered were included as independent variables in a binary logistic regression analysis. The coding of the independent variables is presented in Table 1. The overall model was statistically significant (Omnibus *χ*^2^ = 43.687, df = 11, *p* < 0.001). The Hosmer–Lemeshow goodness-of-fit test indicated an adequate model fit (*χ*^2^ = 3.204, df = 7, *p* = 0.866).

Multivariable analysis showed that participants who had not previously avoided providing first aid were more likely to be classified into C1. First-aid training experience was associated with profile membership. Compared with those who had not received any training, participants who had attended training three or more times were more likely to be assigned to C1, whereas no statistically significant differences were observed for those who had attended training once or twice (both *p* > 0.05). In addition, whether willingness to participate in training would increase if certificates or academic credits were offered was associated with profile membership. Using “significantly increase” as the reference category, participants reporting that their willingness would “slightly increase” were less likely to be classified into C1, whereas those reporting that it would “not change” were more likely to be classified into C1. Sex, age, medical-related educational background, household registration, and parental employment in healthcare-related occupations were not significantly associated with profile membership (all *p* > 0.05) (Table 4).

**Table 4:** Univariable and multivariable binary logistic regression analyses of latent profile membership among ethnic minority freshmen.

Variable	Category	Crude OR (95% CI)	*P*-value	Adjusted OR (95% CI)	*P*-value
Sex	Male	Reference		Reference	
	Female	1.259 (0.840–1.885)	0.265	1.234 (0.810–1.881)	0.327
Age	<18 years	Reference		Reference	
	18–24 years	0.805 (0.376–1.724)	0.576	0.840 (0.380–1.857)	0.666
Medical-related educational background	Yes	Reference		Reference	
	No	0.809 (0.503–1.302)	0.382	0.901 (0.543–1.497)	0.688
Household registration	Urban	Reference		Reference	
	Rural	0.692 (0.451–1.063)	0.093	0.710 (0.450–1.119)	0.140
Parents engaged in healthcare-related occupations	Yes	Reference		Reference	
	No	0.992 (0.401–2.455)	0.986	1.287 (0.490–3.376)	0.609
Previous avoidance of providing first aid	Yes	Reference		Reference	
	No	5.589 (1.342–23.288)	0.018	4.862 (1.156–20.440)	0.031
Whether willingness to participate in training would increase if certificates/academic credits were offered	Significantly increase	Reference		Reference	
	Slightly increase	0.531 (0.316–0.891)	0.017	0.568 (0.335–0.962)	0.036
	No change	3.308 (1.509–7.252)	0.003	3.228 (1.437–7.251)	0.005
First-aid training experience	No training	Reference		Reference	
	Attended training once	1.125 (0.627–2.017)	0.694	0.993 (0.543–1.815)	0.982
	Attended training twice	1.419 (0.812–2.477)	0.219	1.248 (0.699–2.229)	0.453
	Attended training ≥3 times	2.544 (1.664–3.891)	<0.001	2.225 (1.435–3.451)	<0.001

The dependent variable was latent profile membership, coded as 1 = C1 C1 represents the high-attitude/high-practice profile and was coded as 1; C2 represents the low-attitude/low-practice profile and was coded as 0. C2 was the reference profile. OR, odds ratio; CI, confidence interval.

## 4 Discussion

### 4.1 Principal findings

First aid-related KAP among ethnic minority freshmen in this study was not uniformly favorable, which is broadly consistent with previous findings among Chinese university students and freshmen (6, 7, 16). Among the three domains, attitude showed the highest score, whereas transformed knowledge had the lowest score, and self-reported practice remained lower than attitude. This pattern suggests that favorable attitudes may not necessarily be accompanied by sufficient knowledge or self-reported readiness to act in first-aid situations (5, 17). More importantly, the latent profile analysis revealed clear heterogeneity within this population: most students were classified into the low-attitude/low-practice profile, while transformed knowledge scores differed only slightly between profiles. These findings suggest that subgroup differences were mainly reflected in attitudes and self-reported practices rather than factual knowledge alone. Therefore, first-aid education for ethnic minority freshmen should not focus solely on knowledge transmission but should also pay attention to confidence, willingness to act, and perceived readiness to provide first aid. This may be particularly relevant in settings where prior access to structured first-aid education is uneven (18).

### 4.2 Training experience and latent profile membership

Previous first-aid training experience was an important factor associated with latent profile membership. Students who had attended first-aid training three or more times were more likely to belong to the high-attitude/high-practice profile than those without prior training. This finding supports the practical value of repeated exposure to first-aid education and suggests that one-time training may be insufficient to shape more favorable first-aid-related attitudes and self-reported practices (19, 20). Importantly, the value of training may lie not only in exposure itself but also in its continuity. Previous studies have shown that repeated training can improve both CPR performance and willingness to provide help among university students (21). These findings suggest that universities may consider moving beyond one-off sessions and providing regular, skills-based first-aid training across the academic year, particularly for students with less favorable first-aid-related attitudes and self-reported practices. Students with stronger interest, confidence, or motivation toward first aid may also have been more likely to participate in repeated training. Therefore, future longitudinal or intervention studies are needed to further determine whether repeated first-aid training can improve students’ attitudes, confidence, and self-reported practices over time.

### 4.3 Certificate- or credit-related willingness and training preference

In this study, students’ expectations of receiving a certificate or academic credit for first aid training were associated with latent profile membership. Compared with students who reported that certificates or academic credits would significantly increase their willingness to attend training, those who reported that such incentives would not change their willingness were more likely to belong to the high-attitude/high-practice profile, whereas those who reported that their willingness would only slightly increase were less likely to belong to this profile. This pattern may indicate differences in students’ dependence on external incentives rather than a simple motivational effect of certificates or credits. A study of freshmen in Wuhu found that practice lagged behind knowledge and attitudes, highlighting the need for more skills-oriented training (5). In addition, qualitative research among undergraduate medical students identified reward mechanisms as one component of engagement, alongside intrinsic motives such as a voluntary spirit and self-achievement (22). Consistent with this, the American Heart Association scientific statement on resuscitation education highlights the importance of learner-centered educational strategies, including repeated practice, contextual relevance, and training approaches tailored to learners’ needs, in order to improve learning outcomes and behavioral readiness (23). These findings suggest that certificate- or credit-related willingness may reflect differences in students’ training preferences rather than a simple motivational effect of external incentives. Formal recognition, such as certificates, co-curricular credit, or public acknowledgement, may be used as a supplementary strategy to encourage participation, but it should be combined with practical, accessible, and skills-based first-aid training. Such strategies may be particularly useful for students with less favorable first aid-related attitudes and self-reported practices or limited previous exposure to first-aid education.

### 4.4 Previous avoidance of providing first aid and possible response barriers

In this study, students with a tendency to avoid helping were more likely to belong to the lower-level profile. This may reflect barriers beyond knowledge alone, including low confidence to act, fear of making mistakes or causing harm, and uncertainty about possible legal consequences. Previous research among university students has shown that lack of confidence, fear of harming the victim, and fear of legal liability may discourage helping behavior in emergency situations (16). More broadly, a systematic review found that willingness to provide emergency help is associated with training, attitudes, self-efficacy, and perceptions of legal protection, whereas fear of litigation and fear of injuring the victim remain common barriers (24). From a medico-legal perspective, Good Samaritan protections may also be relevant beyond resuscitation alone, including other emergency-helping situations (25). In addition, simulation-based training has been shown to improve not only knowledge and skills but also self-confidence, which may be particularly relevant for students who hesitate to help in real situations (26). However, this interpretation should remain cautious because the number of students who reported previous avoidance was small, and the confidence interval for this association was wide. Moreover, the study did not assess the reasons for avoidance or non-avoidance, nor did it measure legal literacy, perceived legal protection, or fear of legal responsibility. Therefore, medico-legal concerns should be discussed only as a possible contextual factor rather than a directly measured determinant. Future studies should examine specific barriers to first-aid response, including confidence, perceived risk, legal knowledge, and perceived responsibility.

### 4.5 Implications for university first-aid education

Our findings support a shift from sporadic first aid education toward a tiered, equity-oriented training system in universities: ① universal baseline training for all students; ② targeted reinforcement for students in the low-attitude/low-practice profile; and ③ opportunities for advanced peer-support roles to strengthen norms and diffusion. Evidence from China has highlighted both progress and continuing challenges in first aid education, suggesting that university-based systems can serve as a key platform to improve population-level preparedness. In ethnic minority settings, culturally responsive delivery (plain-language materials, culturally relevant scenarios, and, where appropriate, multilingual support) may further enhance acceptability and effectiveness. At the same time, because this study did not objectively assess first-aid skills or real-life helping behavior, educational implications should be understood as suggestions for improving first-aid-related attitudes and self-reported practices rather than as direct evidence of improved emergency response performance.

## 5 Conclusion

This study provides a person-centered understanding of first aid-related KAP among ethnic minority freshmen in Guizhou Province, China. The latent profiles mainly reflected differences in attitudes and self-reported practices rather than substantial differences in transformed knowledge scores, suggesting that first-aid preparedness cannot be understood through knowledge level alone. Previous non-avoidance of providing first aid, repeated first-aid training, and certificate- or credit-related training willingness were associated with membership in the high-attitude/high-practice profile. These findings support the development of targeted university first-aid education that combines repeated practice, scenario-based learning, confidence-building support, and appropriate supplementary recognition. Future intervention studies are needed to examine whether such strategies can improve students’ emergency response confidence, self-reported practices, and objectively assessed first-aid performance.

### 5.1 Strengths and limitations

This study has several strengths. First, it focused on ethnic minority freshmen, a population that has received limited attention in first-aid education research. Second, the relatively large sample allowed the use of a person-centered analytical approach to identify heterogeneity in first aid-related KAP patterns. Third, the use of LPA provided information beyond average group-level scores and helped identify subgroups that may benefit from more targeted first-aid education.

Several limitations should be acknowledged. First, this was a single-university cross-sectional study; therefore, the findings may not be generalizable to all ethnic minority university students, and causal inferences cannot be made. Because military-training company identifiers were not retained, intraclass correlation and multilevel modeling could not be examined. Second, the study relied on self-administered questionnaires, and the practice domain reflected self-reported behavioral intentions rather than objectively assessed first-aid performance. Third, although supplementary EFA and CFA were conducted, the relatively high RMSEA value suggests that the structural validity evidence should be interpreted cautiously and further validated with a refined instrument in independent samples. Fourth, the regression analysis was exploratory. The wide confidence interval for previous non-avoidance of providing first aid may reflect the small number of participants who reported previous avoidance, and this estimate should be interpreted cautiously pending replication in larger samples.

## Data Availability

The raw data supporting the conclusions of this article will be made available by the authors, without undue reservation.
