# Supplementary material for: KAP related to first aid among first-year ethnic minority students: a cross-sectional study using latent profile analysis
Source: Front Public Health. 2026 Jul 23;14:1873238. doi: 10.3389/fpubh.2026.1873238 (PMC13442529; doi:10.3389/fpubh.2026.1873238)
Supplement: Supplementary file 1 [file Table_1.docx]

**Supplementary Table S1. Structural validity analyses of the questionnaire**

| Analysis | Sample | Indicator | Result |
| --- | --- | --- | --- |
| Exploratory factor analysis | Subset A (n = 387) | KMO | 0.889 |
|  |  | Bartlett’s test of sphericity | P < 0.001 |
|  |  | Number of factors extracted | 3 |
|  |  | Cumulative variance explained | 69.58% |
|  |  | Communalities | 0.514–0.849 |
| Confirmatory factor analysis | Subset B (n = 418) | Estimator | DWLS |
|  |  | CFI | 0.994 |
|  |  | TLI | 0.993 |
|  |  | RMSEA | 0.101 (95% CI: 0.092–0.111) |
|  |  | SRMR | 0.065 |

Note: KMO = Kaiser–Meyer–Olkin; CFI = comparative fit index; TLI = Tucker–Lewis index; RMSEA = root mean square error of approximation; SRMR = standardized root mean square residual; DWLS = diagonally weighted least squares.
